# Correlation between *In Vivo* Biofilm Formation and Virulence Gene Expression in *Escherichia coli* O104:H4

**DOI:** 10.1371/journal.pone.0041628

**Published:** 2012-07-25

**Authors:** Rim Al Safadi, Galeb S. Abu-Ali, Rudolph E. Sloup, James T. Rudrik, Christopher M. Waters, Kathryn A. Eaton, Shannon D. Manning

**Affiliations:** 1 Department of Microbiology and Molecular Genetics, Michigan State University, East Lansing, Michigan, United States of America; 2 Center for Food Safety and Applied Nutrition, U.S. Food and Drug Administration, Laurel, Maryland, United States of America; 3 Michigan Department of Community Health, Bureau of Laboratories, Lansing, Michigan, United States of America; 4 Department of Microbiology and Immunology, University of Michigan Medical School, Ann Arbor, Michigan, United States of America; Beijing Institute of Microbiology and Epidemiology, China

## Abstract

The emergence of novel pathogens poses a major public health threat causing widespread epidemics in susceptible populations. The *Escherichia coli* O104:H4 strain implicated in a 2011 outbreak in northern Germany caused the highest frequency of hemolytic uremic syndrome (HUS) and death ever recorded in a single *E. coli* outbreak. Therefore, it has been suggested that this strain is more virulent than other pathogenic *E. coli* (e.g., *E. coli* O157:H7). The *E. coli* O104:H4 outbreak strain possesses multiple virulence factors from both Shiga toxin (Stx)-producing *E. coli* (STEC) and enteroaggregative *E. coli* (EAEC), though the mechanism of pathogenesis is not known. Here, we demonstrate that *E. coli* O104:H4 produces a stable biofilm *in vitro* and that *in vivo* virulence gene expression is highest when *E. coli* O104:H4 overexpresses genes required for aggregation and exopolysaccharide production, a characteristic of bacterial cells residing within an established biofilm. Interrupting exopolysaccharide production and biofilm formation may therefore represent effective strategies for combating future *E. coli* O104:H4 infections.

## Introduction

The 2011 *E. coli* O104:H4 outbreak strain caused the highest frequency of hemolytic uremic syndrome (HUS) and deaths ever recorded. The number of HUS cases and deaths was 2.4 and 1.4 times higher in the *E. coli* O104:H4 outbreak, respectively, than the number reported for 350 *E. coli* O157:H7 outbreaks between 1982 and 2001 in the U.S. These 350 *E. coli* O157:H7 outbreaks resulted in 8,598 cases, 354 cases of HUS and 40 deaths in the U.S. [1], while over 3,816 cases were reported in the 2011 *E. coli* O104:H4 outbreak, with 845 cases of HUS and 54 deaths [2]. The underlying mechanism behind the apparent increase in O104:H4 virulence is not known, though several bacterial factors have been implicated.

The *E. coli* O104:H4 outbreak strain harbors multiple virulence genes from both Shiga toxin (Stx)-producing *E. coli* (STEC) and enteroaggregative *E. coli* (EAEC). It carries the phage-borne gene encoding Stx2, the primary virulence factor of STEC and enterohemorrhagic *E. coli* [3]. Stx2 targets globotriaosyl ceramide receptors in the kidney that contribute to HUS and death in some cases [4]. O104:H4 also possesses the EAEC virulence plasmid (pAA) carrying genes for type I aggregative adherence fimbriae (AAF) [5] that mediate colonization [6] and biofilm formation [7]. AAF expression is induced by AggR, which was implicated in the global regulation of EAEC virulence by controlling expression of a dispersin (*aap*) that allows movement across the intestinal mucosa [8], and an ABC transporter system used for dispersin export [9]. O104:H4 also produces the *Shigella* enterotoxin 1 (ShET1) and Pic serine protease [5], two chromosomally encoded EAEC virulence factors. ShET1 induced fluid accumulation in rabbit ileal loops [10], while Pic facilitated intestinal colonization by enabling the bacterium to utilize mucus as a growth substrate [11]. Because this combination of factors has not previously been observed in pathogenic *E. coli*, the role that they play in colonization and pathogenesis is not known.

Here, we compared the virulence and level of colonization between the *E. coli* O104:H4 outbreak strain and an *E. coli* O157:H7 outbreak strain *in vivo* using germ-free mice and quantified virulence gene expression post-infection. These data enhance our understanding of the mechanism used by the *E. coli* O104:H4 outbreak strain to colonize the host and contribute to toxin-mediated disease.

## Results

### *E. coli* O104:H4 Colonization in Germ-free Mice

Five mice were inoculated with 8×10^6^ CFU of O104:H4 and euthanized seven days post inoculation (PI). Clinical presentation, colonization density, cecal weight, and hisopathology data were compared to the five mice inoculated with 1.0×10^6^ CFU of O157:H7 five days PI. Several clinical signs including lethargy, weight loss, dehydration, and low urine specific gravity, a measure of reduced kidney function, were observed in O157:H7-infected mice; all mice developed renal acute tubular necrosis (ATN). O104:H4-infected mice displayed fewer signs of infection with no signs of renal ATN seven days PI.

**Figure 1:**
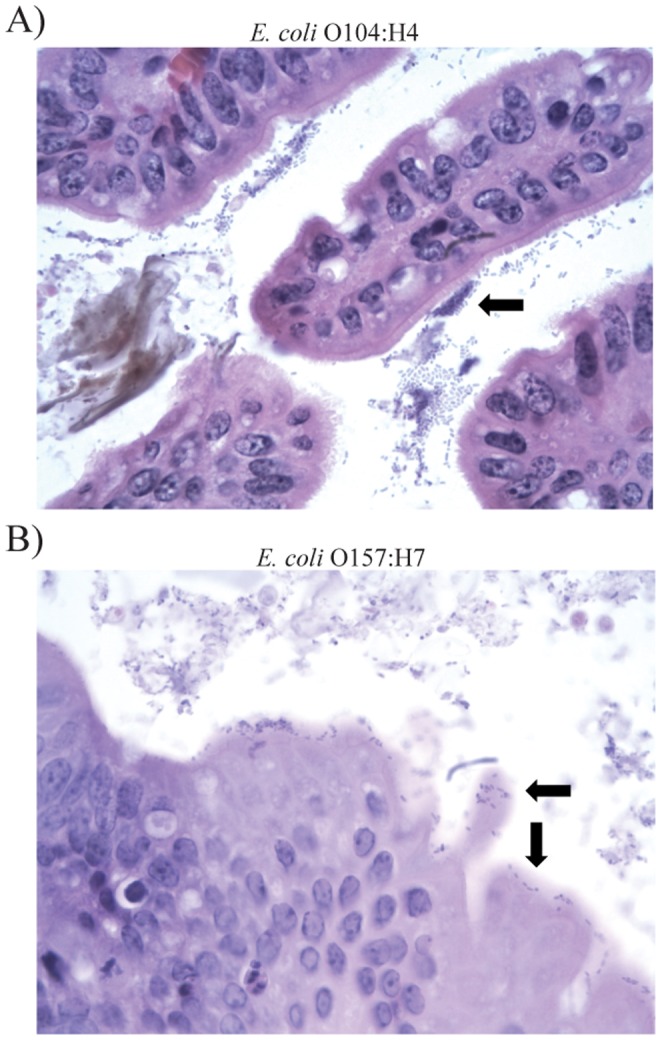
Histological examination of cecal tissue. Arrows highlight A) bacterial aggregation associated with the mucus layer in the cecum of *E. coli* O104:H4-infected mouse 11–272, and B) bacteria associated with the cecal mucosa in *E. coli* O157:H7-infected mouse 11–437. Cells were stained with hematoxylin and eosin and magnified 1000x.

Interestingly, the cecal colonization density was significantly higher in O104:H4-infected mice seven days PI (mean: 4.4×10^11^ CFU/g) versus O157:H7-infected mice five days PI (mean: 1.67×10^10^) (*P* = 0.016). It is possible, however, that the difference in colonization density between the mice infected with the same strain is due to varying infection periods (Fig. S1). Histological examination of the cecum demonstrated bacterial aggregation in O104:H4-infected mice that was not apparent in O157:H7-infected mice (Fig. 1). Based on this finding, we hypothesized that the aggregative adherence observed at seven days PI represents the early stage of a biofilm.

### Biofilm Formation by *E. coli* O104:H4

To determine whether O104:H4 expresses genes important for biofilm formation *in vivo*, we quantified expression of *aggR* and *pic* that are unique to O104:H4. No difference in *pic* expression was observed *in vivo* at seven days PI relative to *in vitro* planktonic growth, whereas *aggR* was 13.3 fold lower *in vivo* than *in vitro* (Table S1). Gene expression was also examined for *pgaA*, the first gene in the *pgaABCD* operon that is responsible for poly-beta-1,6-N-acetyl-D-glucosamine (PGA) synthesis in *E. coli* [12]. At seven days PI, the overall level of *pgaA* expression was similar in O104:H4 relative to O157:H7 at five days (Table S2). Differences in expression between the two strains, however, were observed *in vivo* versus planktonic growth *in vitro*. The average level of *pgaA* expression in O104:H4 was 5.0 fold higher *in vivo*, while no significant difference was observed for O157:H7.

**Figure 2:**
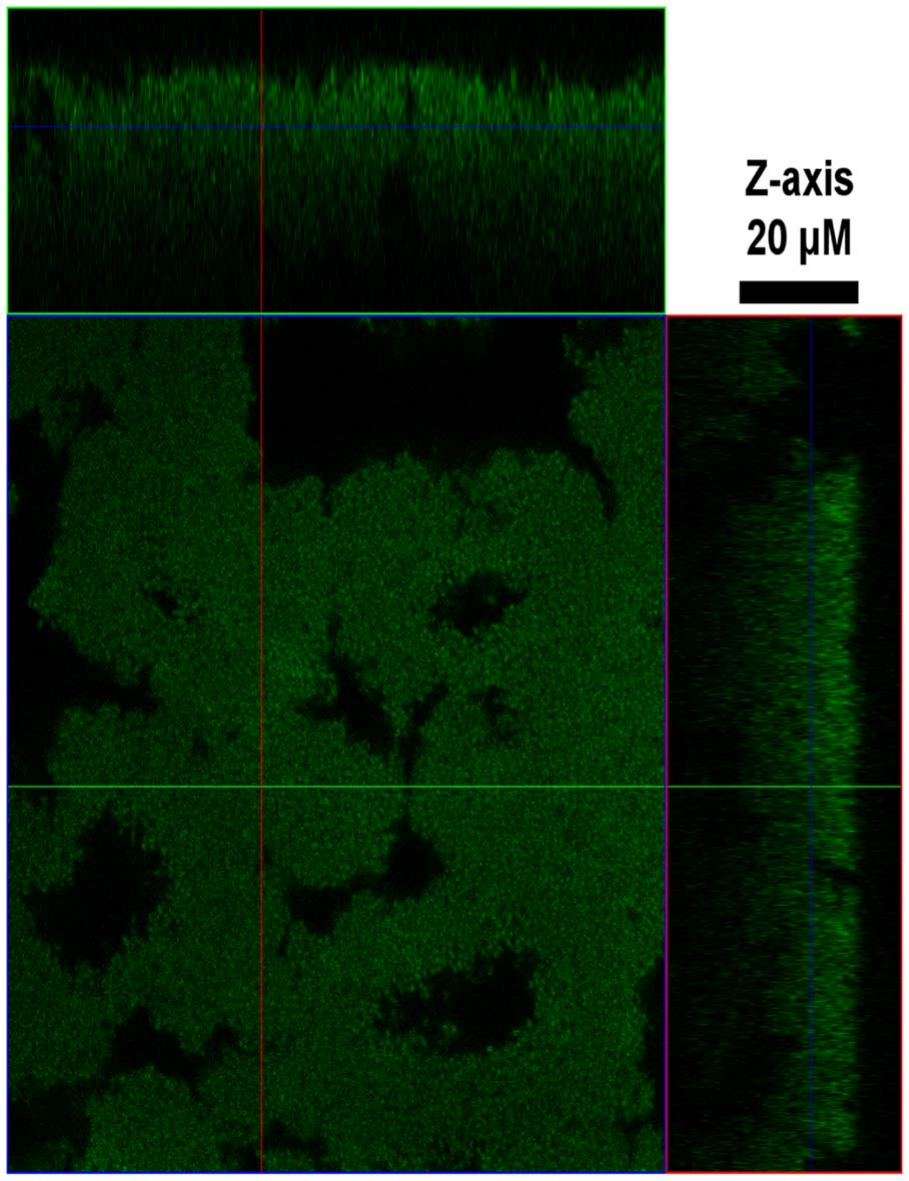
*E. coli* O104:H4 forms thick biofilms *in vitro*. Confocal laser scanning microscopy revealed the presence of microcolonies (green) following growth of *E. coli* O104:H4 (TW16133) for 16 hours in a flow cell. Black space represents conduits or channels used for nutrient transport within the biofilm, while the z-axis denotes the thickness.

Importantly, O104:H4 also exhibited robust biofilm formation *in vitro* in both static and flow based assays. For the static assay, biofilms were allowed to form on the sides and bottom of the wells of a microtiter plate. After removal of planktonic cells, biofilms were stained with crystal violet followed by quantification of retained dye by measuring the optical density (OD) at 595 nm. O104:H4 had a 7.3-fold greater level of biofilm formation compared with O157:H7 in this assay (1.33+/−0.19 versus 0.18+/−0.02, respectively). Growth of O104:H4 in a flow cell for 16 hours resulted in a 20 µm biofilm (Fig. 2) with complex structures including microcolonies and water channels that permit nutrient and oxygen transport into the biofilm. These data further demonstrate that the O104:H4 outbreak strain readily forms biofilms when grown in non-planktonic conditions that favor biofilm formation; such conditions do not support biofilm formation in O157:H7.

### *E. coli* O104:H4 and Renal Disease in Germ-free Mice

Two additional groups of five mice were inoculated with O104:H4 for 13–15 days to increase the likelihood of biofilm formation *in vivo*. The groups received 2.8×10^6^ CFU and 1.0×10^7^ CFU of O104:H4 and were euthanized 15 and 13 days PI, respectively. Two mice died one day PI, but the remaining eight mice became colonized at comparable levels in the cecum (Fig. S1). Unlike seven days PI, a lengthier infection resulted in renal ATN in six of the eight mice, though considerable variation in the severity of the kidney lesions was observed across mice (Table S3). Because Stx2 production contributes to renal ATN in mice [13], it is likely that variation in disease severity is due to the differential production of Stx2 *in vivo*.

### *In vivo* Expression of Bacterial Genes

**Figure 3:**
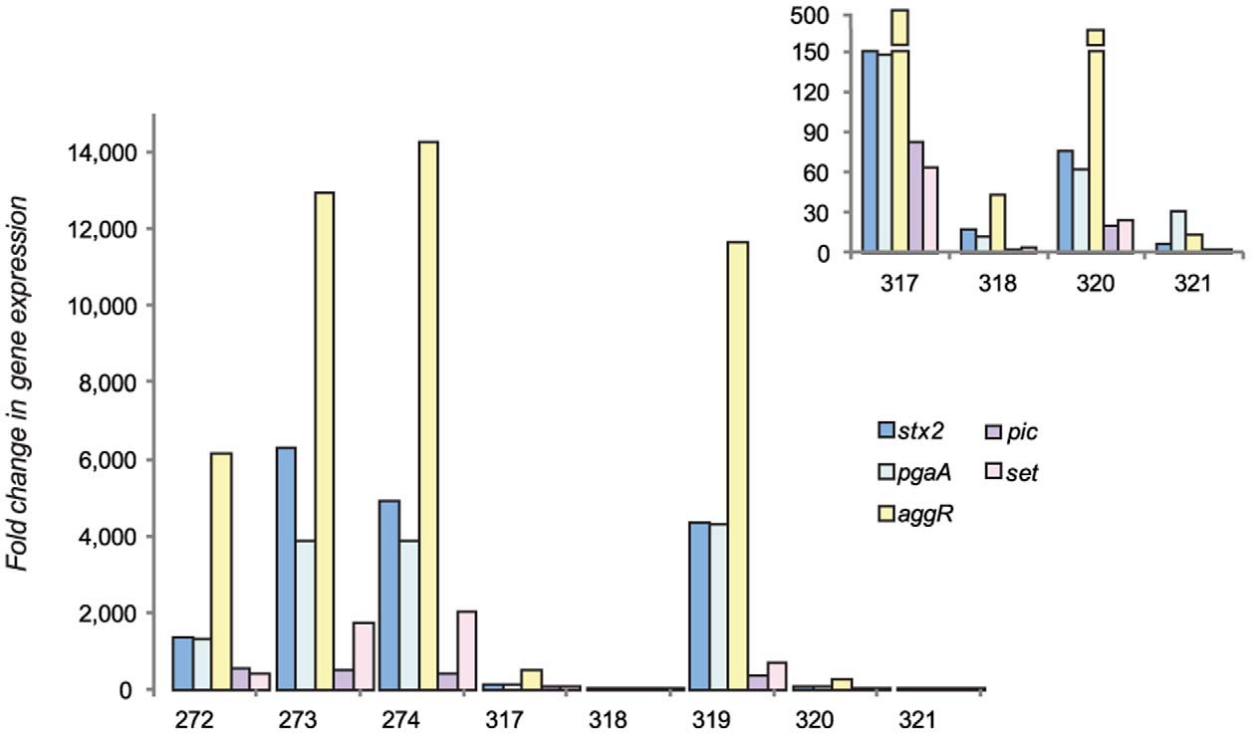
Variation in bacterial gene expression among mice challenged with *E. coli* O104:H4 for 13–15 days. Y-axis represents the fold change in cecal gene expression levels among eight mice infected with *E. coli* O104:H4 at 13–15 days post infection relative to mice infected for seven days. The inset highlights the four mice with fold change values that cannot be visualized using the y-axis in the larger histogram. Broken bars indicate a large change in the y-axis value, while the x-axis refers to the mouse identification number.

*In vivo stx2* expression, which represents a marker for Stx2 production [14], was quantified to examine its relationship with disease severity. There was considerable variation in *stx2* induction between mice at each time point. Average *in vivo stx2* expression in O104:H4 was 2.3 fold lower than in O157:H7 at seven and five days PI (Table S2). The lengthier infection resulted in an average 2,146-fold increase in *stx2* expression for O104:H4 at 13–15 versus seven days PI (Fig. 3, Table S3). It is therefore possible that the increased and prolonged expression of *stx2* may partly explain the high frequencies of HUS observed during the O104:H4 outbreak.

**Figure 4:**
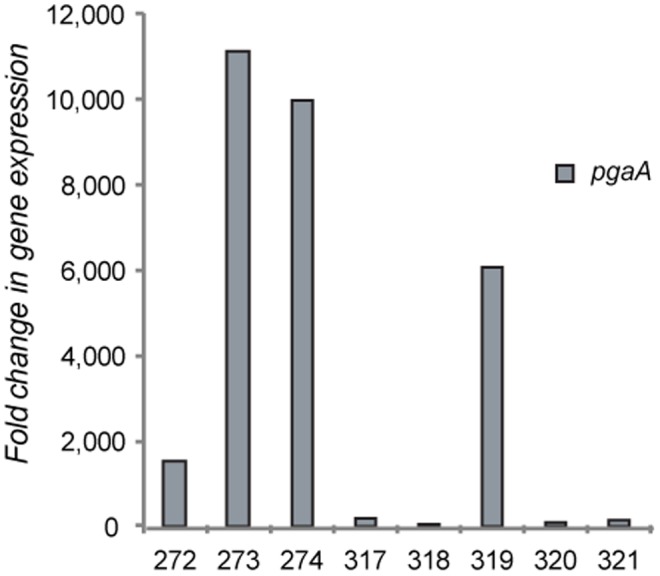
Variation in *pgaA* expression among mice challenged with *E. coli* O104:H4 for 13–15 days relative to planktonic growth *in vitro*. The Y-axis represents the fold change in cecal gene expression levels while the x-axis is the mouse identification number.

*In vivo stx2* expression was highly correlated with expression of both *aggR* (Pearson correlation coefficient (CC): 0.97; *P*<0.0001) and *pgaA* (CC: 0.97; *P*<0.0001), but there was no correlation between expression and cecal colonization density. Average induction of *aggR* increased 5,721-fold (range: 13.7- to 14,247.2-fold) at 13–15 versus seven days PI, while *pgaA* increased 1,700-fold (range: 12- to 4, 301-fold). Furthermore, *pgaA* expression was on average 3,640-fold higher (range: 23.6- to 11,141.7-fold) at 13–15 days PI relative to *in vitro* growth (Fig. 4, Table S4). Collectively, these data suggest that a stable biofilm with high levels of exopolysaccharide production forms *in vivo*, as determined by *pgaA* expression, and is correlated with both *aggR* and *stx2* expression.

A significant increase in gene expression was also observed for *pic* and *set*, which encodes ShET1, between seven and 13–15 days PI (Fig. 3). Average induction of *pic* was 245-fold (2.8- to 553.2-fold) higher at 13–15 days PI relative to seven days, while *set* increased 626-fold (2.7- to 2,032-fold). Comparable expression levels were observed for all four virulence genes at 13–15 days PI relative to growth *in vitro*, though the fold change was lower than what was observed for 13–15 versus seven days PI (Table S4). Like *stx2*, expression of *pic* (CC: 0.85; *P* = 0.008) and *set* (CC: 0.93; *P* = 0.0005) were highly correlated with *aggR* at 13–15 days PI as well as *pgaA*, though the correlation was not as strong. These data suggest that both the host environment and biofilm formation, which is likely due to *pgaA* expression followed by *aggR* induction, play an important role in the transcription of other O104:H4 virulence genes.

### Gene Expression and Disease Severity

Expression of *aggR* was also correlated with disease severity in mice based on the extent of renal ATN (CC: 0.70; *P* = 0.05). The two mice with an over 12,000-fold increase in *aggR* expression, for instance, had the highest levels of *stx2* expression (Fig. 3) and most extensive renal necrosis (Table S2). Mouse number 11.272, however, had high levels of both *aggR* and *stx2* but less extensive renal necrosis. Weight and age at inoculation, colon necrosis score, cecum weight, cecal colonization density, and weight loss were not correlated with *aggR* expression or renal ATN, suggesting that Stx2 production is most important for kidney disease development.

## Discussion

The results described herein provide insight into the mechanism of *E. coli* O104:H4 pathogenesis. Specifically, formation of a biofilm *in vivo* contributes to enhanced virulence gene expression and an increased likelihood of kidney damage. These observations were not noted for mice infected with *E. coli* O157:H7, which colonized in single layers displaying the characteristic pattern of intimate attachment to the epithelia [15], [16] via genes encoded on the locus of enterocyte effacement (LEE) [17]. The *E. coli* O104:H4 outbreak strain, however, lacks the LEE but has the pAA [5], which is important for aggregative adherence and biofilm formation [7]. The *in vivo* aggregation of O104:H4 in germ free mice was similar to the aggregative adherence patterns observed previously in HEp-2 cells for O104:H4 [5] and other EAEC strains [18]. Similar to observations of EAEC infections in gnotobiotic piglets [19], O104:H4 aggregation also appeared to be associated with the mucus layer, thereby lending support to the idea that the mechanism of pathogenesis differs considerably between the two strain types.

Consistent with prior studies, mice infected with O157:H7 developed kidney lesions by five days PI [13], [20]. By contrast, kidney lesions were not observed in O104:H4-infected mice until 13–15 days PI. These findings support the observation that *E. coli* O157:H7 contributes to rapid onset of disease in mice [13], [20] and support reports that the incubation period in humans for O157:H7 and O104:H4 infections is four and eight days, respectively [2], [21]. The lengthier incubation period of O104:H4 is consistent with reports suggesting that biofilm-associated infections have longer incubation periods [22].

Although there was evidence of O104:H4 biofilms *in vivo* at seven days PI, the level of *pgaA* expression increased by up to 4,301-fold at 13–15 days PI suggesting that an extensive biofilm had formed over the two week period. Prior studies have demonstrated that the PGA exopolysaccharide, the primary component of the self-produced extracellular matrix of a biofilm, is critical for maintaining the structure of biofilms [12]. Our data suggest that the O104:H4 strain expresses genes important for biofilm formation during *in vivo* infection but that O157:H7 does not. Because PGA facilitates the transition from temporary to permanent bacterial attachment [23], increased *in vivo pgaA* expression at seven days also supports the hypothesis that biofilm formation is an important first step in *E. coli* O104:H4 pathogenesis. In addition, a 14,247-fold average increase in expression of *aggR*, a global regulator of virulence genes, was observed *in vivo* at 13–15 versus seven days PI. This delayed induction of *aggR* suggests that conditions do not necessarily favor biofilm gene activation in germ-free mice prior to seven days PI. Once induced, however, AggR may initiate a positive feedback loop that results in its own amplification [24], a mechanism that may partly explain the marked increase in *aggR* expression levels observed at 13–15 days PI. This increased expression of *aggR* likely results in enhanced production of AAF, which contribute to biofilm formation [7]. Such coordinated expression of virulence genes is another example of concerted regulation of virulence to produce a ‘balanced pathogenicity’ effect, as was recently described in enteropathogenic *E. coli* [25].

Based on these findings, we hypothesize that O104:H4 biofilms begin to form in germ-free mice by seven days PI and that these biofilms are capable of producing low levels of exopolysaccharide. The production of exopolysaccharide likely induces *aggR*, which was repressed *in vivo* at seven days PI relative to planktonic growth *in vitro*, and contributes to *aggR* autoactivation and the subsequent production of AAF. Over time, enhanced fimbriae production and other colonization factors likely contribute to the formation of a more stable biofilm capable of excreting high levels of exopolysaccharide. Future mutagenesis studies, however, are needed to directly test the role of both *aggR* and *pgaA* in biofilm formation and *E. coli* O104:H4 pathogenesis.

It is important to note that there was considerable variation in bacterial gene induction across mice as well as a wide range in gene expression levels. Nonetheless, expression of all five genes was significantly higher in all mice infected with O104:H4 for 13–15 days PI relative to mice infected for seven days and *in vitro.* The higher level of induction at 13–15 days PI relative to seven days PI and to *in vitro* growth suggests that specific signals within the host play an important role in virulence gene expression. The observed correlation between *aggR*, *pgaA* and *stx2* is also noteworthy because induction of *stx2* is important for renal ATN development. Two mice with an over 12,000-fold increase in *aggR* expression *in vivo* at 13–15 versus seven days PI, for example, also had a 4,900-fold increase in *stx2* expression. While there was a correlation between *aggR* expression and ATN, this correlation was likely driven by the two mice with the largest increase in *aggR* and *stx2* expression over time. One mouse, 11.272, showed markedly high levels of *aggR* and *stx2*, but had a low ATN score. It is possible that in this mouse transcription of *stx2* did not correlate with toxin production, which was demonstrated in prior studies [14], [26], or that the transmission of Stx2 across the intestinal barrier differed. It is also possible that there is a threshold in mice such that gene expression over a certain value does not linearly correlate with disease outcome. Nevertheless, these data suggest that the factors influencing biofilm formation and disease progression likely vary across hosts.

Like humans, individual mice colonized by the same bacterial strain differed in clinical presentation. Such differences may be due to variation in immune and inflammatory responses and in the type of microbial communities, nutrients and environmental cues available in the gastrointestinal tract. Because germ-free mice lack resident microbiota and have an immature immune response, it is likely that variation in host susceptibility is due to innate responses impacting the ability of bacteria to communicate with each other (quorum sensing) and/or to form biofilms *in vivo*. Survival within a biofilm protects bacteria from host defenses and decreases the effectiveness of antibiotics [27], which is due in part to nutrient limitation responses [28]. Limited nutrient availability was previously shown to enhance *stx2* expression in *E. coli* O157:H7 [26] and may explain the observed correlation between *aggR* and *stx2* expression. Moreover, the type I AAF of EAEC were shown to induce host inflammatory responses [29] and disrupt epithelial cell barrier function [30], which may more readily permit the penetration of toxins to the intestinal submucosa and facilitate their absorption.

Although most of these bacterial responses represent strategies for *E. coli* O104:H4 to survive in the complex host environment, they are inadvertently enhancing bacterial virulence. This increased virulence that results from survival within a biofilm may have implications for other biofilm-forming, toxin-producing pathogens as well. Importantly, several mice were colonized with high levels of O104:H4, but in the absence of markers demonstrating significant biofilm formation, the severity of these infections was reduced. Interrupting exopolysaccharide production and biofilm formation could represent an effective strategy that can be used to combat future *E. coli* O104:H4 infections.

## Materials and Methods

### Ethics Statement

*E. coli* O104:H4, strain TW16133, was isolated by a clinical microbiology laboratory in Michigan from a patient infected during travel to Germany and deposited into the Michigan Department of Community Health (MDCH) strain collection. Because STEC is a notifiable disease in the state of Michigan, informed consent was not obtained by the patient. The Institutional Review Board of Michigan State University and the MDCH approved the study protocol. The isolate was anonymized and stripped of all patient identifiers before use in the experiments described herein. Approval to use germ-free mice was granted by the University Committee on Use and Care of Animals at the University of Michigan in accordance with guidelines from the Association for Assessment and Accreditation of Laboratory Animal Care International whose regulations are based on the Guide for the Care and Use of Laboratory Animals by the National Institutes of Health.

### Bacterial Strains

Following isolation, the *E. coli* O104:H4, strain TW16133, was confirmed to be the outbreak strain via pulsed field gel electrophoresis, *stx* profiling, and pAA detection. *E. coli* O157:H7 EDL933 [31] was used for comparison.

### Murine Challenge Experiments

Germ free Swiss-Webster mice, 3–4 weeks of age, were obtained from the University of Michigan breeding colony where they are maintained in soft-sided bubble isolators and are free of exposure to all bacterial, fungal, viral, and parasitic organisms. Mice were aseptically transferred to sterile microisolator cages and housed in a sterile laminar flow hood; both the food and water given to the mice were sterile.

Ten mice were inoculated via oral gavage with O157:H7 or O104:H4 as described [13] and euthanized at five and seven days PI, respectively. Surgical procedures were performed under sodium pentobarbital anesthesia in order to minimize suffering. All mice were bacteriologically sterile, except for being colonized with O104:H4, as was verified by terminal aerobic and anaerobic cultures as well as Gram stains of feces and cecal contents. Mice infected with O157:H7 were euthanized earlier than O104:H4-infected mice because all mice became moribund by five days PI. Eight additional O104:H4-infected mice were euthanized at 13 or 15 days PI. Bacterial colonization (cfu/g cecal contents) was quantified by plate dilution on LB agar. Following formalin fixation and histological examination of cecal and kidney tissues, the extent of renal ATN was scored as follows: no tubular necrosis (0); necrotic tubules rarely present (1); necrotic tubules in many fields (2); and necrotic tubules in most fields (3). Tissues were blinded for scoring. Colonization densities of O157:H7- and O104:H4-infected mice were compared using the Mann-Whitney U test (two-tailed).

### Biofilms

Isolates were grown overnight in LB and diluted (OD_600_ = 0.05) in DMEM with 0.45% glucose. Tissue culture plates were inoculated and incubated aerobically at 37°C for 16 hours, washed, and stained with crystal violet for 10 min. Wells were washed and dried, and the crystal violet was solubilized with ethanol for 45 min. The biofilm biomass was estimated at OD_595_; all assays were performed in quadruplicate, repeated three times and the standard deviation was calculated. For growth in a flow cell, O104:H4 was inoculated (OD_600_ = 0.05) and exposed to DMEM at 0.5 ml/min for 16 hours at 37°C as described [32]. Cells were fixed, stained with 20 µM Syto 9, and visualized using an Axioskop2 microscope (Zeiss).

### Bacterial Gene Expression

To quantify gene expression *in vivo*, bacterial RNA was isolated from murine cecal contents using a modified hot-phenol extraction protocol [33] and purified with the RNeasy Mini kit (Qiagen). For *in vitro* expression, strains were grown in DMEM with glucose and NaHCO_3_ (OD_600_ = 0.65) prior to RNA extraction. cDNA was synthesized by reverse transcription as highlighted in our prior study [34]. Three independent RNA populations were extracted per sample/strain and cDNA was generated from the same amount of RNA (1 ug) from each sample to avoid bias that may be introduced by differences in bacterial colonization densities that can proportionally affect the amount of RNA. qPCR was performed in triplicate utilizing six primer sets (Table S5); the 16 S rRNA gene, *rrsH*, was used for normalization and transcription differences were calculated as described [34]. Fold changes ≥2 were considered biologically significant.

## Supporting Information

Figure S1 **Cecal colonization levels (cfu/g of cecal contents) in germ-free mice infected with** ***E. coli*** **O104:H4 at 7 days and 13–15 days post infection (PI) relative to mice infected with** ***E. coli*** **O157:H7.**
(TIF)

Table S1 **Fold-change differences in gene expression in germ-free mice infected with** ***E. coli*** **O104:H4 seven days post infection relative to** ***E. coli*** **O104:H4** ***in vitro*** **growth.**
(DOC)

Table S2 **Fold-change differences in gene expression in germ-free mice infected with** ***E. coli*** **O104:H4 at 7 days post infection relative to mice infected with** ***E. coli*** **O157:H7 at 5 days post infection.**
(DOC)

Table S3 **Fold-change differences in** ***E. coli*** **O104:H4 gene expression** ***in vivo*** **13–14 days post infection as compared to seven days PI and acute tubular necrosis (ATN) scores.**
(DOC)

Table S4 **Relative differences in** ***E. coli*** **O104:H4 gene expression** ***in vivo*** **13–14 days post infection as compared to growth** ***in vitro*** **.**
(DOC)

Table S5 **Primers used for quantitative RT-PCR.**
(DOC)
