# Supplementary figures and images for: Correlation between In Vivo Biofilm Formation and Virulence Gene Expression in Escherichia coli O104:H4
Source: PLoS One. 2012 Jul 25;7(7):e41628. doi: 10.1371/journal.pone.0041628 (PMC3405000; doi:10.1371/journal.pone.0041628)

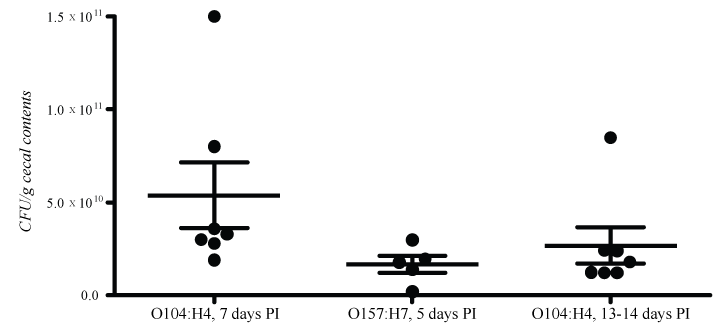

Supplement: Figure S1 — Cecal colonization levels (cfu/g of cecal contents) in germ-free mice infected with E. coli O104:H4 at 7 days and 13–15 days post infection (PI) relative to mice infected with E. coli O157:H7. (TIF) [file pone.0041628.s001.tif]
