# Supplementary material for: Correlation between In Vivo Biofilm Formation and Virulence Gene Expression in Escherichia coli O104:H4
Source: PLoS One. 2012 Jul 25;7(7):e41628. doi: 10.1371/journal.pone.0041628 (PMC3405000; doi:10.1371/journal.pone.0041628)
Supplement: Table S1 — Fold-change differences in gene expression in germ-free mice infected with E. coli O104:H4 seven days post infection relative to E. coli O104:H4 in vitro growth. (DOC) [file pone.0041628.s002.doc]

**Table S1.** Fold-change differences in gene expression in germ-free mice infected with *E. coli* O104:H4 seven days post infection relative to *E. coli* O104:H4 *in vitro* growth.

|  | **Quantitative RT-PCR (fold change)** | | | | |
| --- | --- | --- | --- | --- | --- |
| **Mouse number** | ***pga*** | ***stx2*** | ***aggR*** | ***pic*** | ***set*** |
| 11.238 | 5.3 | -2.5 | -10.0 | -1.7 | -2.3 |
| 11.239 | 7.8 | -1.4 | -33.3 | -1.0 | -1.7 |
| 11.240 | 3.5 | 1.5 | -3.3 | -1.0 | -2.0 |
| 11.241 | 2.0 | -1.0 | -10.0 | 1.0 | -2.4 |
| 11.242 | 6.6 | -2.1 | -10.0 | -1.1 | -3.0 |

Positive and negative fold-change values indicate induction or repression of gene expression in mice infected with O104:H4 seven days PI as compared to growth *in vitro*.
