# Supplementary material for: Correlation between In Vivo Biofilm Formation and Virulence Gene Expression in Escherichia coli O104:H4
Source: PLoS One. 2012 Jul 25;7(7):e41628. doi: 10.1371/journal.pone.0041628 (PMC3405000; doi:10.1371/journal.pone.0041628)
Supplement: Table S2 — Fold-change differences in gene expression in germ-free mice infected with E. coli O104:H4 at 7 days post infection relative to mice infected with E. coli O157:H7 at 5 days post infection. (DOC) [file pone.0041628.s003.doc]

**Table S2.** Fold-change differences in gene expression in germ-free mice infected with *E. coli* O104:H4 at 7 days post infection relative to mice infected with *E. coli* O157:H7 at 5 days post infection.

|  | **Quantitative RT-PCR (fold change)** | |  |
| --- | --- | --- | --- |
| **Mouse number** | ***pga*** | ***stx2*** | |
| 11.238 | -2.0 | -3.0 | |
| 11.239 | -1.6 | -2.2 | |
| 11.240 | 1.2 | -1.4 | |
| 11.241 | -1.5 | -1.8 | |
| 11.242 | -1.9 | -3.2 | |

Positive and negative fold-change values indicate induction or repression of gene expression in mice infected with O104:H4 seven days PI as compared to mice infected with O157:H7.
