# Supplementary material for: Correlation between In Vivo Biofilm Formation and Virulence Gene Expression in Escherichia coli O104:H4
Source: PLoS One. 2012 Jul 25;7(7):e41628. doi: 10.1371/journal.pone.0041628 (PMC3405000; doi:10.1371/journal.pone.0041628)
Supplement: Table S3 — Fold-change differences in E. coli O104:H4 gene expression in vivo 13–14 days post infection as compared to seven days PI and acute tubular necrosis (ATN) scores. (DOC) [file pone.0041628.s004.doc]

**Table S3**: Fold-change differences in *E. coli* O104:H4 gene expression *in vivo* 13-14 days post infection as compared to seven days PI and acute tubular necrosis (ATN) scores.

|  | **Quantitative RT-PCR (fold change)** | | | | |  |
| --- | --- | --- | --- | --- | --- | --- |
| **Mouse number** | ***pga*** | ***stx2*** | ***aggR*** | ***pic*** | ***set*** | **ATN** |
| 11.272 | 1292.3 | 1358.9 | 6138.0 | 553.2 | 425.5 | 1 |
| 11.273 | 3878.4 | 6294.9 | 12919.6 | 510.7 | 1740.1 | 3 |
| 11.274 | 3857.5 | 4900.2 | 14247.2 | 408.5 | 2032.7 | 3 |
| 11.317 | 147.9 | 149.7 | 517.0 | 81.9 | 62.9 | 0 |
| 11.318 | 12.0 | 17.6 | 43.0 | 3.1 | 3.9 | 2 |
| 11.319 | 4301.2 | 4363.9 | 11626.4 | 380.6 | 718.1 | 2 |
| 11.320 | 62.4 | 76.2 | 263.7 | 20.5 | 24.2 | 2 |
| 11.321 | 31.5 | 6.7 | 13.7 | 2.8 | 2.7 | 2 |

Fold change differences in gene expression *in vivo* 13-14 days post infection (PI) relative to *in vivo* seven days PI expression levels in *E. coli* O104:H4 strain, TW16133. ATN was considered to be present in mice with a kidney lesion score of ≥ 2, with 3 representing the highest score possible.
