# Supplementary material for: Correlation between In Vivo Biofilm Formation and Virulence Gene Expression in Escherichia coli O104:H4
Source: PLoS One. 2012 Jul 25;7(7):e41628. doi: 10.1371/journal.pone.0041628 (PMC3405000; doi:10.1371/journal.pone.0041628)
Supplement: Table S4 — Relative differences in E. coli O104:H4 gene expression in vivo 13–14 days post infection as compared to growth in vitro . (DOC) [file pone.0041628.s005.doc]

**Table S4**: Relative differences in *E. coli* O104:H4 gene expression *in vivo* 13-14 days post infection as compared to growth *in vitro*.

|  | | **Quantitative RT-PCR (fold change)** | | | | | | | | | | |
| --- | --- | --- | --- | --- | --- | --- | --- | --- | --- | --- | --- | --- |
| **Mouse number** | | ***pga*** | | ***stx2*** | | ***aggR*** | | ***pic*** | | ***set*** | |  |
| 11.272 | 1,532.0 | | 623.9 | | 795.2 | | 239.9 | | 184.2 | |  | |
| 11.273 | 11,142.7 | | 2,855.7 | | 1,582.5 | | 397.8 | | 1,174.2 | |  | |
| 11.274 | 9,967.3 | | 2,738.9 | | 2,667.5 | | 390.9 | | 699.5 | |  | |
| 11.317 | 178.0 | | 76.7 | | 38.2 | | 99.9 | | 27.0 | |  | |
| 11.318 | 23.6 | | 8.0 | | 2.8 | | 3.0 | | 1.8 | |  | |
| 11.319 | 6,045.0 | | 1,077.7 | | 786.7 | | 279.5 | | 306.5 | |  | |
| 11.320 | 115.4 | | 33.8 | | 17.6 | | 18.2 | | 10.3 | |  | |
| 11.321 | 119.7 | | 5.2 | | 2.1 | | 2.1 | | 1.8 | |  | |

Fold change differences in gene expression *in vivo* 13-14 days post infection (PI) in *E. coli* O104:H4 strain TW16133 relative to *in vitro* expression levels.
