# Supplementary material for: Correlation between In Vivo Biofilm Formation and Virulence Gene Expression in Escherichia coli O104:H4
Source: PLoS One. 2012 Jul 25;7(7):e41628. doi: 10.1371/journal.pone.0041628 (PMC3405000; doi:10.1371/journal.pone.0041628)
Supplement: Table S5 — Primers used for quantitative RT-PCR. (DOC) [file pone.0041628.s006.doc]

**Table S5.** Primers used for quantitative RT-PCR.

| **Primer** | **Sequence (5’-3’)** | **Gene** | **Amplicon size (bp)** |
| --- | --- | --- | --- |
| shet_F551 | CTGTTCCCCCACAGAGTGTT | *shet* | 139 |
| shet_R689 | GTGAACCGACGATTGGAAAT |
|  |  |  |  |
| pic_F3696 | CCTGACAGAGGACACGTTCA | *pic* | 147 |
| pic_R3842 | TCAACCCCTGTTCTTCCAAC |
|  |  |  |  |
| pgaA_F377 | CTCTGGAGCCGCAAAATAAG | *pgaA* | 140 |
| pgaA_R516 | GGCTTCTGCGAGTAAATTGG |
|  |  |  |  |
| aggR_F486 | TTCCGATAAGGTCAGAAACACA | *aggR* | 169 |
| aggR_R654 | TGCTGCTTTGCTCATTCTTG |
|  |  |  |  |
| stx2B_F | GAAGATGTTTATGGCGGT | *stx* | 115 |
| stx2B_R | CACTGTAAATGTGTCATC |
|  |  |  |  |
| rrsH_F | CGATGCAACGCGAAGAACCT | *16S rRNA* | 178 |
| rrsH_R | CCGGACCGCTGGCAACAAA |
